# GLP-1 receptor agonists and pancreatic beta cell apoptosis in diabetes mellitus: a systematic review and meta-analysis of preclinical studies

**DOI:** 10.3389/fcdhc.2025.1579961

**Published:** 2025-08-27

**Authors:** Nicolas Rea, Prakash V. A. K. Ramdass

**Affiliations:** Department of Public Health and Preventive Medicine, St. George’s University School of Medicine, St. George, Grenada

**Keywords:** diabetes mellitus, GLP-1 receptor agonists, GLP-1RAs, pancreatic beta cell, apoptosis, exenatide, exendin-4

## Abstract

**Introduction:**

Diabetes mellitus (DM) is a global health challenge characterized by progressive beta cell dysfunction. Glucagon-like peptide-1 receptor agonists (GLP-1RAs) have emerged as promising therapies, enhancing insulin secretion while potentially preserving beta cell mass by inhibiting apoptosis. However, concerns persist regarding long-term beta cell adaptation and functional exhaustion. This meta-analysis synthesizes preclinical evidence to evaluate the effects of GLP-1RAs on beta cell apoptosis in DM.

**Methods:**

Following PRISMA guidelines, we systematically searched Scopus, PubMed, Embase, and Google Scholar for preclinical studies assessing GLP-1RAs effects on human beta cell apoptosis. Five studies met inclusion criteria for meta-analysis. Data were extracted on apoptotic rates, and risk of bias was assessed using the OHAT tool. A random-effects model calculated pooled mean differences (MDs) in apoptosis, with sensitivity analyses and funnel plots evaluating robustness and publication bias.

**Results:**

GLP-1RAs significantly reduced beta cell apoptosis (pooled MD: −0.10; 95% CI: −0.15 to −0.05, p = 0.0003), with high heterogeneity (I² = 100%). Sensitivity analyses confirmed consistency, with effect estimates ranging from −0.077 to −0.118 upon sequential study exclusion. Funnel plot and Egger’s test (p = 0.80) indicated no significant publication bias, though limited study numbers constrain power.

**Conclusions:**

GLP-1RAs demonstrate a robust anti-apoptotic effect on pancreatic beta cells in preclinical models, supporting their role in preserving beta cell mass. However, extreme heterogeneity and unresolved questions about long-term functional exhaustion warrant cautious interpretation. Future research should prioritize longitudinal human studies to assess clinical relevance and optimize therapeutic strategies. Introduction

**System review registration:**

https://www.crd.york.ac.uk/PROSPERO/view/CRD42024516313, identifier CRD42024516313.

## 1 Introduction

Diabetes mellitus (DM) is a chronic metabolic disorder characterized by sustained hyperglycemia resulting from either insufficient insulin secretion, impaired insulin action, or both. Globally, approximately 6.1% of the population—around 529 million people—live with DM, a number expected to rise due to aging populations, sedentary lifestyles, and increasing obesity rates (1). Effective management of DM remains a major clinical challenge, despite advances in hypoglycemic therapies (2). Among newer pharmacologic options, glucagon-like peptide-1 receptor agonists (GLP-1RAs) have emerged as a promising class of drugs that enhance glucose-dependent insulin secretion while offering potential benefits beyond glycemic control (3).

GLP-1RAs exert their effects by binding to receptors on pancreatic beta cells, activating intracellular signaling pathways that amplify insulin release in response to hyperglycemia (4). Beyond their insulinotropic effects, growing evidence suggests that GLP-1RAs may play a critical role in preserving beta cell function by inhibiting apoptosis and promoting beta cell proliferation (5). These mechanisms could help sustain beta cell mass and delay the progressive decline in function characteristic of type 2 DM. However, the long-term implications of GLP-1RAs therapy remain debated.

While preclinical and clinical studies highlight their anti-apoptotic and proliferative effects, concerns persist that prolonged receptor activation might induce beta cell adaptation or “functional exhaustion,” where chronic stimulation could diminish intrinsic secretory capacity over time (6). This paradox underscores the need to clarify whether GLP-1RAs provide durable protection or pose risks of beta cell dependency (7). For instance, some studies report that GLP-1RAs reduce oxidative stress and apoptosis, thereby preserving beta cell mass (8, 9), whereas others suggest that sustained signaling may downregulate endogenous pathways, impairing beta cell responsiveness (10).

Given these conflicting perspectives, this systematic review and meta-analysis aims to synthesize preclinical evidence on the effects of GLP-1RAs on beta cell apoptosis in DM, providing a clearer understanding of their potential benefits and limitations in preserving beta cell health.

## 2 Materials and methods

### 2.1 Study protocol registration

We conducted a systematic review and meta-analysis according to the Preferred Reporting Items for Systematic Reviews and Meta-Analyses (PRISMA) guidelines (11). Our study protocols were registered in the PROSPERO International prospective register of systematic reviews (registration ID CRD42024516313).

### 2.2 Search strategy and selection criteria

Database searching was completed using the following search queries in Scopus, PubMed, Embase, and Google Scholar: (“glucagon-like peptide-1 receptor agonists” OR “GLP-1 agonists” OR “GLP-1RAs”) AND (“beta cell apoptosis”) AND (“diabetes mellitus”). We also searched references for additional studies. Citation files from each database were imported into Zotero reference management software and duplicates were removed. Two reviewers (N.R. and P.R.) first screened the titles and abstracts independently based on the selection criteria, and the full text of studies that met the eligibility criteria were further screened for inclusion. Any disagreements were resolved through discussion. The selection criteria for this review were limited to published, original, peer reviewed pre-clinical studies that investigated the effects of GLP-1RAs on apoptosis from pancreatic beta cell cultures obtained from humans with diabetes mellitus. Our search included studies from inception to June 20, 2025. Studies were restricted to the English language and publication type as “article.” We excluded animal studies, case reports, conference proceedings, abstracts, and reviews.

### 2.3 Data extraction

Data extraction was performed by two reviewers (N.R., and P.R.) using a standardized form, capturing the study author and year of publication, study location, study design, cell count, apoptotic agent, detection method of apoptosis, and the percentage of apoptotic cells in the GLP-1RAs treatment group and the control group. The standard deviation (SD) was calculated using the formula: SD = sqrt (P * (1-P)/n), where sqrt is the square root; P is the proportion of apoptosis; and n is the cell count. Data was extracted from the results and relevant tables. In some instances where the raw data was unavailable, data was extrapolated from figures.

### 2.4 Data analysis and publication bias

All statistical tests were considered significant if p < 0.05. All statistical analyses were performed using R statistical software (version 4.4.3; R Foundation for Statistical Computing, Vienna, Austria) with the metafor package for meta-analytic computations. The primary outcome was the mean difference (MD) in beta cell apoptosis between the GLP-1RAs-treated group and the control group. Forest plots were generated to visualize individual study estimates and the pooled effect size with 95% confidence intervals (CIs). We used a random-effects model due to high heterogeneity across preclinical studies, as assessed via the I² statistic and its corresponding p-value.

To assess the robustness of our findings, we conducted a leave-one-out sensitivity analysis, systematically excluding each study to evaluate its influence on the overall effect estimate.

To estimate potential missing studies, we visually inspected the funnel plot for symmetry. Objective testing with Egger’s regression (12) was performed, despite the limited number of included studies.

### 2.5 Risk of bias assessment

Risk of bias (quality) assessment was carried out by two reviewers (N.R., and P.R.) using the Office of Health Assessment and Translation (OHAT) Risk of Bias Rating Tool for Human and Animal Studies, evaluating the following 9 metrics: randomization of dose/exposure, allocation concealment, confounding, blinding, incomplete outcome data, selective reporting, sequence generation, selective outcome reporting, and other sources of bias (13).

## 3 Results

### 3.1 Study characteristics

**Figure 1:**
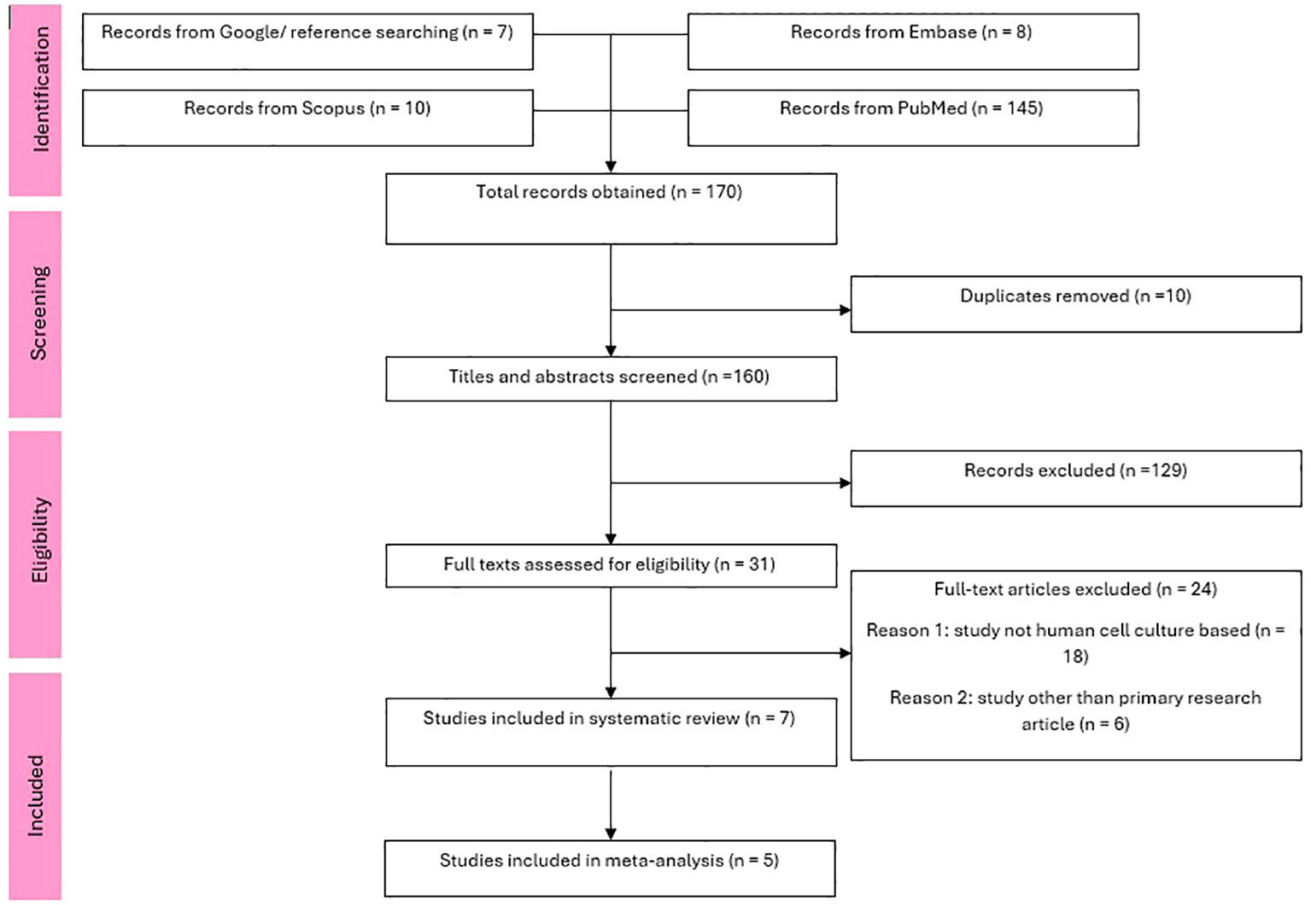
PRISMA flow chart of included studies.

Our search across 4 databases and citation searching yielded a total of 201 studies. After duplicates were removed, 160 abstracts and titles were screened and 31 articles met eligibility for full text screening. After further screening a total of 7 studies (14–20) met inclusion criteria for the systematic review and 5 studies (14–18) for the meta-analysis. A PRISMA flow chart of the details is shown in **Figure 1**.

The systematic review included seven preclinical studies investigating the effects of GLP-1RAs on pancreatic beta cell apoptosis, while the meta-analysis included four studies. Key characteristics of these studies are summarized in **Table 1**. Studies originated from North America (n=4) and Europe (n=3), reflecting diverse countries yet limited preclinical evidence from only two geographical regions. Varied GLP-1RAs were tested, including native GLP-1 (n=1), exendin-4 (n=3), and exenatide (n=2). Cell counts ranged from 100 to 2,800 beta cells per study (with half of the cells in the treatment group and half in the control group), with apoptosis induced by agents such as palmitate (n=3), oleate/tert-butyl hydroperoxide (t-BHP) (n=1), prohuman islet amyloid polypeptide (prohIAPP) (n=1), and cytokines (n=1). One study by Farilla et al. (14) measured natural cell death without exogenous apoptotic stimuli. Fluorescence microscopy (n=3) and cell death ELISA (n=2) were most common in detecting beta cell apoptosis.

**Table 1:** Characteristics of included studies.

Study	Country	GLP-1RA	Cell count	Apoptotic agent/method	Detection method of apoptosis
Farilla, 2003 (14)	USA	GLP-1	400	Natural cell death	Fluorescence microscopy
Buteau, 2004 (15)	Canada	GLP-1	400	Palmitate	Fluorescence microscopy
Liu, 2012 (16)	USA	Exendin-4	1000	Oleate, t-BHP	ATPlite assay
Park, 2013 (17)	Canada	Exenatide	2400	prohIAPP	Chemiluminescence microscopy
Cunha, 2014 (18)	Belgium	Exendin-4	100	Palmitate	Fluorescence microscopy
Systematic review only					
Natalicchio, 2013 (19)	Italy	Exenatide	2800	Palmitate	Cell death detection ELISA
Varin, 2016 (20)	France	Exendin-4	1000	Cytokines (Tpl2 inhibitor)	Cell Death Detection ELISA

GLP-1, glucagon-like peptide-1; GLP-1RA, glucagon-like peptide-1 receptor agonist; t-BHP, tert-butyl hydroperoxide; Tpl2 inhibitor, tumor progression locus 2; prohIAPP, prohuman islet amyloid polypeptide.

The two studies in the systematic review only highlight the protective effects of GLP-1RAs on beta-cell apoptosis under stress conditions but focus on different mechanisms. Varin et al. (20) demonstrated that inhibition of the MAP3 kinase Tpl2 in beta cells protects against cytokine-induced apoptosis and dysfunction, with enhanced efficacy when combined with the GLP-1RA exendin-4. This combination suppressed proinflammatory pathways (ERK1/2, JNK, p38) and preserved insulin secretion in rodent and human islets. Natalicchio et al. (19) showed that exendin-4 counteracts palmitate-induced beta-cell apoptosis by downregulating GPR40 expression and inhibiting MKK4/7-mediated activation of stress kinases (JNK, p38) via a PKA-dependent mechanism. Together, these findings underscore the potential of GLP-1RAs to mitigate beta-cell apoptosis through distinct anti-inflammatory and metabolic pathways, offering therapeutic strategies for diabetes by targeting cytokine- or lipotoxicity-driven beta-cell damage.

### 3.2 Meta-analysis

**Figure 2:**
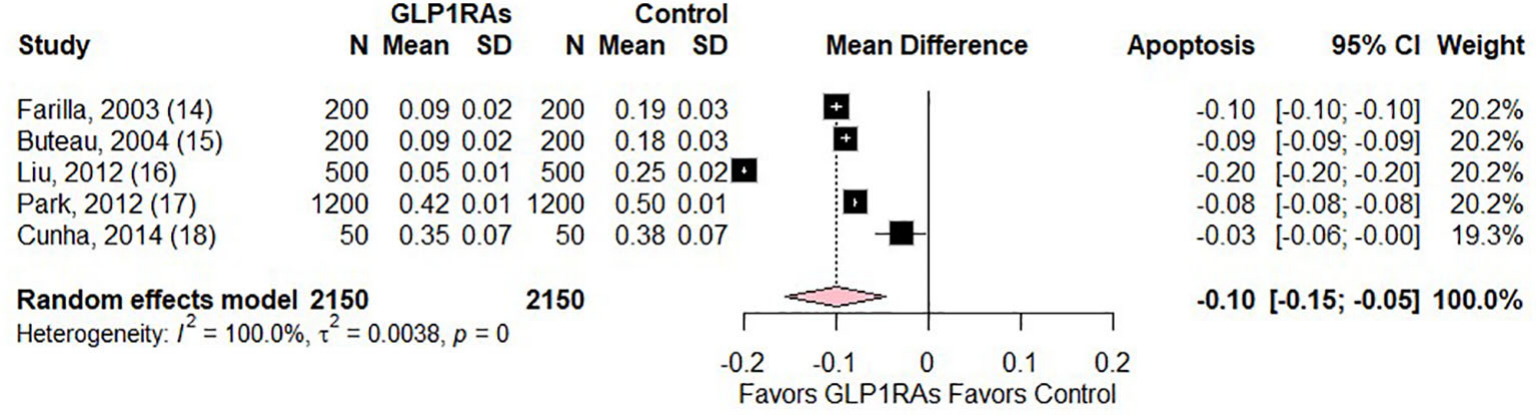
Forest plot of mean difference in apoptosis between GLP-1RAs and control.

The forest plot, depicted in **Figure 2**, presents a meta-analysis comparing the mean difference (MD) in beta cell apoptosis between patients treated with GLP1RAs and controls in DM. Five studies were included, with sample sizes ranging from 50 to 1200 participants. The MD in apoptosis rates consistently favoured GLP1RAs, with values ranging from -0.03 to -0.20, indicating reduced apoptosis in the GLP1RAs group. The random effects model yielded a pooled MD of -0.10 (95% CI: -0.15 to -0.05; p = 0.0003), demonstrating a statistically significant reduction in beta cell apoptosis with GLP1RAs treatment. All individual study results and the overall estimate fell to the left of the null line, further supporting the protective effect of GLP1RAs against beta cell apoptosis. The weights assigned to each study were relatively balanced, contributing to a robust pooled estimate. However, there was significant heterogeneity (I^2^ = 100.0%, p = 0.0).

### 3.3 Sensitivity analysis

**Figure 3:**
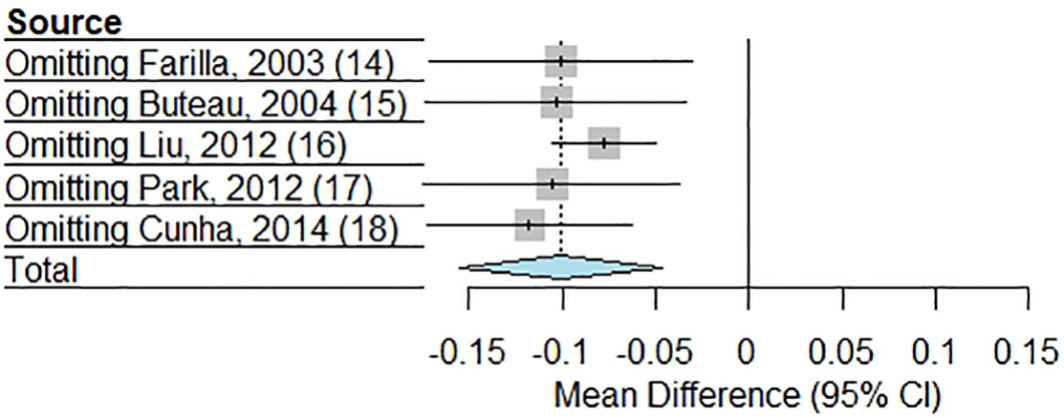
Leave-one-out sensitivity analysis for the meta-analysis.

The sensitivity analysis, shown in **Figure 3**, assessed the robustness of the meta-analysis by systematically omitting each study one at a time to evaluate its influence on the pooled MD in beta cell apoptosis between GLP-1RAs and control groups. The results demonstrated consistency in the overall effect, as the exclusion of any single stud did not substantially alter the direction or significance of the pooled estimate. Sequentially excluding each study yielded effect estimates ranging from -0.077 to -0.118, all remaining statistically significant (p ≤ 0.0049) and directionally consistent with the pooled estimate (-0.101, 95% CI: -0.155 to -0.047). Heterogeneity remained high (I² = 100%) in all analyses except when excluding the study by Liu et al. (I² = 96.7%) (16), suggesting this study contributed modestly to between-study variability. Notably, exclusion of the study by Cunha et al. (18) produced the largest effect magnitude (-0.118), while omitting the study by Liu et al. (16) yielded the most precise estimate (95% CI: -0.105 to -0.050). The consistency of effects across all sensitivity analyses suggests that the meta-analysis findings are not disproportionately driven by any individual study, reinforcing the reliability of the conclusion that GLP-1RAs significantly reduces beta cell apoptosis compared to controls.

### 3.4 Publication bias

**Figure 4:**
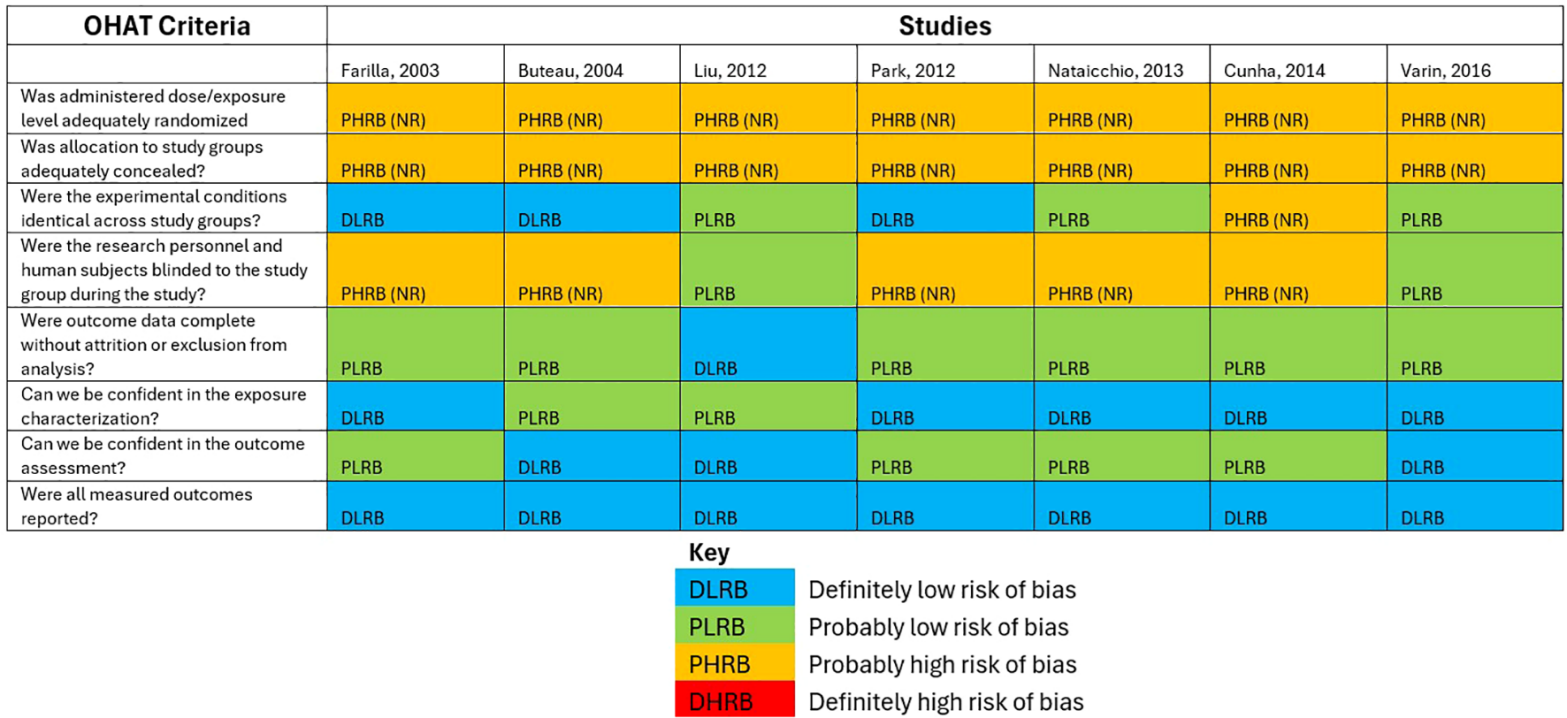
Risk of bias assessment with OHAT tool.

Visual inspection of the funnel plot, depicted in **Figure 4**, does not indicate asymmetry. Further objective testing with Egger’s regression test revealed no significant funnel plot asymmetry (t = -0.28, p = 0.80), suggesting low risk of publication bias. However, it must be noted that the small number of studies limits the power of this test.

### 3.5 Risk of bias assessment

**Figure 5:**
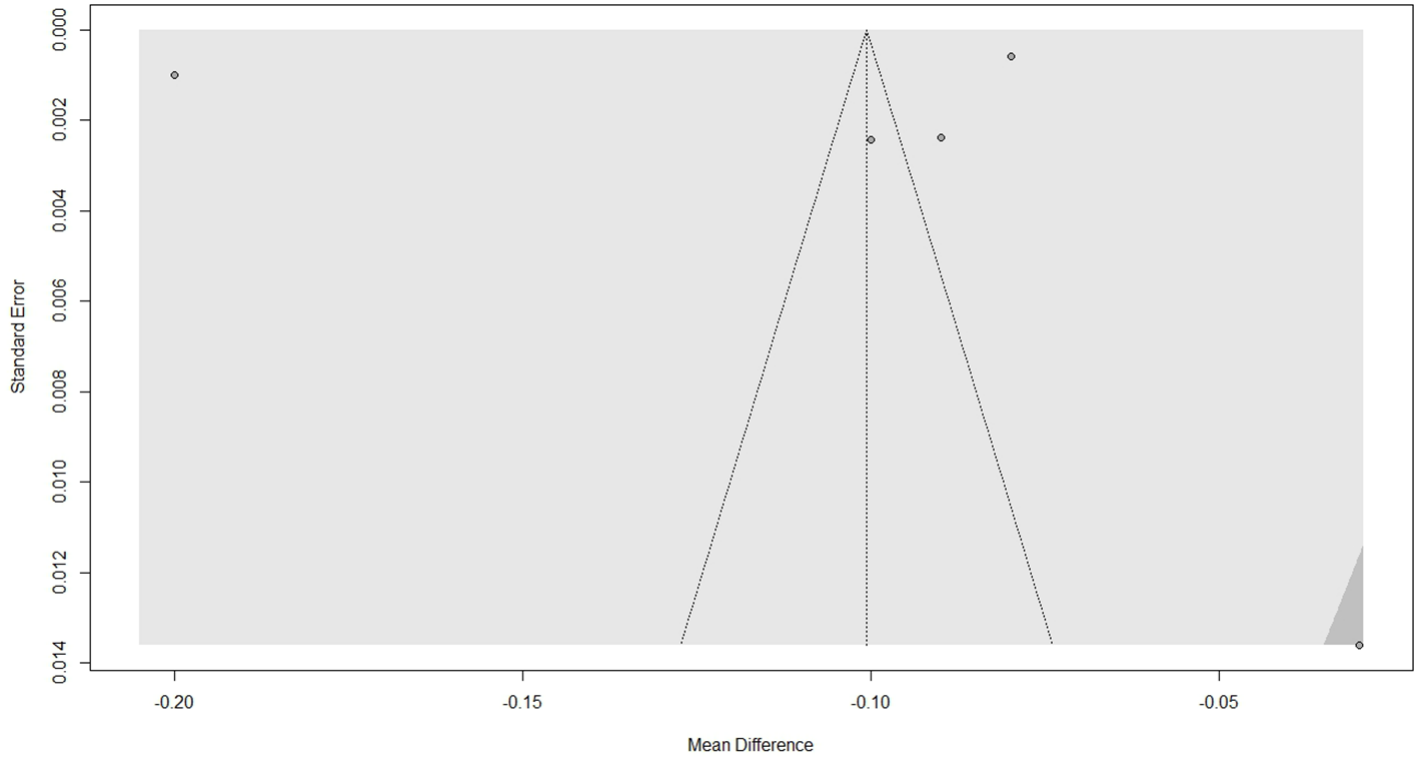
Funnel plot for publication bias.

The risk of bias assessment using the OHAT tool (13) evaluated seven studies investigating the effects of GLP-1RAs on beta cell apoptosis in diabetes mellitus. These findings are shown in **Figure 5**. Key methodological concerns were identified, particularly in randomization and allocation concealment, where most studies were rated as “probably high risk of bias” (PHRB), often due to insufficient reporting (NR). Blinding of research personnel and subjects was also frequently rated as PHRB or “probably low risk of bias” (PLRB), indicating variability in study design rigor. Outcome data completeness was mostly deemed PLRB, suggesting moderate confidence in attrition reporting. However, confidence in exposure characterization and outcome assessment varied, with several studies rated as “definitely low risk of bias” (DLRB) or PLRB, while others raised concerns (DLRB or PLRB for outcome assessment). All studies adequately reported measured outcomes (DLRB). Overall, the assessment highlights inconsistencies in study quality, particularly in randomization and blinding, warranting cautious interpretation of the meta-analysis results.

## 4 Discussion

This systematic review and meta-analysis examined the impact of GLP-1RAs on pancreatic beta cell function and potential exhaustion in patients with diabetes mellitus. While GLP-1RAs are widely used to improve glycemic control and support weight loss, their effects on beta cell health over time remain complex, highlighting both protective and potentially detrimental aspects (21).

Our analysis reveals that GLP-1RAs contribute to the preservation of beta cell function, resulting in 10% reduction in apoptosis when compared to controls. However, the utility of this protective effect is yet to be assessed *in-vivo*, and it is unclear if these effects are clinically relevant. Nevertheless, recent meta-analyses have confirmed that GLP-1RAs are highly efficacious in achieving glycemic control in patients with diabetes mellitus (22, 23). Moreso, several studies demonstrated that GLP-1RAs increase beta cell mass by reducing apoptosis and encouraging cellular proliferation, suggesting a protective mechanism that may slow the natural progression of beta cell dysfunction in type 2 DM (24). One mechanism that explains this preservation in beta cell function is that GLP-1RAs stimulate insulin secretion in a glucose-dependent manner and inhibit glucagon release (25). This glucose-dependency is crucial, as it helps prevent hypoglycemia and minimizes undue beta cell stress when glucose levels are low (26). Additionally, GLP-1RAs stimulate regeneration, which enhances cell survival and function under stress (27). Evidence suggests that GLP-1RAs, such as exenatide and liraglutide, help protect pancreatic beta cells by reducing the apoptosis triggered by lipotoxic and glucotoxic conditions (28). These agonists achieve this by activating beneficial signaling pathways that promote cell survival and reduce inflammation within the pancreatic islets. They work by modulating PI3K/Akt and MAP kinase pathways, leading to reduced production of inflammatory cytokines like TNF-α and IL-1β (29).

In addition, GLP-1RA reduce beta cell apoptosis by modulating inflammatory pathways and promoting anti-apoptotic signaling, contributing to the preservation of beta cell mass (21). Moreover, GLP-1 receptor activation can enhance the expression of factors like PDX-1 and MafA, which are involved in insulin gene expression and beta cell regeneration, further adding to the protection of beta cell mass (30, 31).

Despite these benefits, questions about the potential for beta cell exhaustion with chronic GLP-1RAs use remain unanswered. While the glucose-dependent nature of GLP-1RAs activity suggests a degree of protection against overstimulation, prolonged therapy may create a scenario where beta cells become less responsive to endogenous signals and more reliant on exogenous agonists for insulin production (32). Long-term exposure to GLP-1RAs might induce adaptive changes in beta cells, possibly leading to functional exhaustion due to overreliance on exogenous stimulation (33). Studies indicate that continuous pharmacologic activation of GLP-1 receptors could heighten beta cell activity, potentially exhausting cellular reserves and accelerating dysfunction in susceptible individuals (34). This risk of “functional burnout” is particularly relevant for patients with advanced diabetes or those on high doses of GLP-1RAs.

Given the mixed effects on GLP-1 agonists’ impact on beta cell health, caution may be warranted when considering these agents for long-term, continuous therapy (35). The potential for beta cell exhaustion suggests that individualized treatment approaches are necessary, particularly for patients at different stages of beta cell function decline (36). Some research advocates for intermittent therapy with GLP-1RAs, though more studies are required to assess the efficacy and safety of such protocols in preserving long-term beta cell health (37).

The findings of this review underscore the importance of careful patient monitoring during GLP-1RAs therapy, especially for long-term users. Regular assessment of beta cell function and glycemic control markers may help clinicians detect early signs of beta cell exhaustion and adjust treatment plans accordingly. Three of the studies considered here also only used the short-acting GLP-1RAs exendin-4/exenatide (16–18). However, the long-term effects of prolonged activation of GLP-1 receptors by long-acting GLP-1RAs on beta cell exhaustion remain uncertain, as it is unclear whether sustained stimulation might lead to adaptive changes that could impair beta cell functionality or contribute to eventual cellular exhaustion. However, it has been shown that albiglutide, a long-acting GLP-1RA, has demonstrated effects in preserving beta cell function (albeit just for 1 year), as measured by mixed meal tolerance test and plasma C-peptide levels (38). Thus, it may be of interest to test the long-acting GLP-1RAs such as semaglutide, liraglutide, and dulaglutide (24).

Interestingly, most of the studies in this analysis focused on apoptosis at relatively consistent conditions of exendin paired with palmitate, which has been found to increase the levels of cytotoxicity and apoptosis induced in cells, suggesting that the protective effect may be higher than suggested (26). The mechanism by which palmitate increase apoptosis in cells results from the inhibition of DNA synthesis and the creation of reactive oxygen species (27). Moreover, studies have shown that while rats have a higher number of glucose sensing mechanisms on beta cells, humans have higher expression of radical scavenging systems, and thus possibly further lowering the suspected effectiveness of these models *in-vivo* human experiments (28).

Future studies may also look to assess the effectiveness of GLP-1RAs in different conditions, be it more physiological levels and treatments. The challenge in doing so stems from carrying out these studies in humans can be rather challenging, though previous studied *in-vivo* using mice have yielded positive results, showing increased islet mass and beta cell proliferation with reduced apoptosis when rats were infused with human recombinant GLP-1 (39).These results are promising as the GLP-1RAs directly influence the pancreas very heavily in mice (40), but the task becomes far more challenging in humans as GLP-1RAs have far reaching effects, such as brain derived GLP with function in neurons, and GLP-1 receptor detection in tissues can be a complicated process making it hard to state the effects are due to the agonist itself (10).

### 4.1 Limitations

Several limitations should be considered when interpreting the findings of this systematic review and meta-analysis. First, the small number of included studies (n=7 for the systematic review, n=5 for meta-analysis) limits the statistical power and generalizability of the results. Additionally, the high heterogeneity (I² = 100%) observed in the meta-analysis suggests variability in study designs, apoptotic stimuli, and GLP-1RA formulations, which may complicate direct comparisons. The predominance of studies from North America and Europe also restricts the global applicability of the findings. Methodological concerns were identified in the risk of bias assessment, particularly regarding randomization, allocation concealment, and blinding, which could introduce bias. Furthermore, the exclusive focus on *in vitro* studies raises questions about the translational relevance to human physiology, as cell culture models may not fully replicate the complex *in vivo* diabetic microenvironment. Finally, while Egger’s regression test did not detect publication bias, the small number of studies reduces the reliability of this assessment. These limitations underscore the need for more standardized preclinical studies and future *in vivo* investigations to validate the anti-apoptotic effects of GLP-1RAs.

## 5 Conclusions

In summary, while GLP-1RAs offer significant benefits in managing diabetes and protecting beta cell function, they present a nuanced challenge regarding potential beta cell exhaustion with prolonged use. Clinicians and researchers must weigh the immediate benefits of GLP-1RAs against the potential long-term risks, aiming to optimize therapeutic outcomes while preserving beta cell integrity in diabetes care.

## Data Availability

The original contributions presented in the study are included in the article/Supplementary Material. Further inquiries can be directed to the corresponding author.
